# Comparison of Hemorrhage on CT Versus MRI After Thrombectomy: The HECATE Study

**DOI:** 10.1161/SVIN.124.001441

**Published:** 2024-08-30

**Authors:** Amie W. Hsia, Lawrence L. Latour, Sana Somani, Carolyn A. Lomahan, Yongwoo Kim, John K. Lynch, Marie Luby

**Affiliations:** ^1^ NIH/NINDS, Stroke Branch Bethesda MD; ^2^ MedStar Washington Hospital Center Comprehensive Stroke Center Washington DC

**Keywords:** acute ischemic stroke, CT, endovascular therapy, hemorrhagic transformation, MRI

## Abstract

**Background:**

The characterization of hemorrhage following acute stroke intervention has largely been computed tomography (CT) based. We sought to compare magnetic resonance imaging‐ (MRI‐) and CT‐based scoring of hemorrhage after acute endovascular therapy (EVT) applying the Heidelberg Bleeding Classification (HBC) to assess intermodal agreement and quantify interrater agreement.

**Methods:**

Consecutive patients with acute stroke were included in this retrospective study if they (1) had MRI and CT ≤12 hours of each other OR (2) had CT bracketed by MRI pre‐ and post‐CT (ie, MRI‐CT‐MRI) ≤7 days post‐EVT. The concordance of the HBC ratings by consensus panel were compared between CT and T2^*^ gradient recalled echo MRI.

**Results:**

For the 87 EVT‐treated patients included, median age was 68 years [60–74], admission National Institutes of Health Stroke Scale score 18 [13–23], 47% were treated with intravenous/intraarterial thrombolytics, and 93% were successfully recanalized (modified Thrombolysis in Cerebral Infarction 2b/3). Hemorrhage was detected on at least 1 modality in 60% (52/87) of patients. We found a 68% (59/87, 95% CI [57%–77%]) agreement overall between CT and MRI for hemorrhage classification post‐EVT. MRI had the best interrater agreement for HBC 0 (no hemorrhage) with excellent concordance (ĸ = 0.882), compared with CT (ĸ = 0.683). T2^*^ gradient recalled echo MRI tended to have increased sensitivity to scattered petechial hemorrhage (HBC 1a) as compared with CT with 17% (2/12) intermodal agreement. The interrater agreement of HBC class 2 (ie, parenchymal hematoma grade 2 was substantial for MRI (ĸ = 0.781) and excellent in CT (ĸ = 0.951), with 67% (8/12) intermodal agreement. Subarachnoid hemorrhage was detected in 24% (21/87) of patients on CT and/or MRI with 29% (6/21) intermodal agreement.

**Conclusion:**

With the exception of subarachnoid hemorrhage and minor petechial hemorrhagic transformation, we found that MRI and CT are overall interchangeable for detecting and classifying hemorrhage after EVT. These findings are reassuring for both clinical decision‐making and research application. Given the complexity of hemorrhage subtypes post‐EVT, work to further refine a post‐EVT hemorrhage classification scale with clinical correlation would be beneficial.

**Nonstandard Abbreviations and Acronyms**

ECASS: European Cooperative Acute Stroke Study
EVT: endovascular therapy
FLAIR: fluid attenuated inversion recovery
GRE: gradient recalled echo
HBC: Heidelberg Bleeding Classification
HT: hemorrhagic transformation
ICH: intracranial hemorrhage
PH‐2: parenchymal hematoma grade 2
sICH: symptomatic intracranial hemorrhage

**Clinical Perspective**

**What Is New?**

- In this first comparison of magnetic resonance imaging versus computed tomography to classify hemorrhage after endovascular therapy using the Heidelberg Bleeding Classification, we found a 68% agreement overall between the 2 imaging modalities, including excellent agreement for Heidelberg Bleeding Classification Class 2 (ie, parenchymal hematoma grade 2).
- Discordance between magnetic resonance imaging and computed tomography post‐endovascular therapy occurred in detection of minor petechial bleeding and subarachnoid hemorrhage.

**What Are the Clinical Implications?**

- Results of this study suggest that either magnetic resonance imaging or computed tomography are overall interchangeable for detecting and classifying hemorrhage after endovascular therapy for acute ischemic stroke.

Reperfusion therapy for acute ischemic stroke, particularly with expanding indications, applications, and devices for endovascular therapy (EVT), has been rapidly increasing in recent years.^1^ With the increase in acute stroke intervention, including in extended time windows and in the setting of larger core ischemic lesions, there has been an increased frequency of hemorrhagic transformation (HT) and other intracranial hemorrhage (ICH) including intraventricular and subarachnoid hemorrhage (SAH).^2^, ^3^ To more effectively categorize post‐EVT HT and ICH than prior classification scales such as that developed by the European Cooperative Acute Stroke Study (ECASS) group for intravenous thrombolysis, the Heidelberg Bleeding Classification (HBC) was developed with input from representatives of the 5 initial positive endovascular acute ischemic stroke trials published in 2015.^4^, ^5^ Although the HBC was designed with the explicit intention to apply to both CT and MRI post‐EVT, the HBC authors acknowledged that the HT categories are computed tomography (CT) based and that a comparison of the different types of HT by the 2 imaging modalities has not been well studied.^4^

Categorization of HT and/or ICH has implications for future clinical reperfusion or neuroprotectant trials that may become increasingly magnetic resonance imaging (MRI) based, including for patient selection, patient outcomes, and determining investigational device or drug safety and efficacy. It is also common clinical practice to obtain a post‐EVT CT or MRI scan, often at 24 hours, to assess infarct size and presence of HT or ICH to guide prognosis and clinical management, for example, timing of antiplatelet or anticoagulation initiation. When hemorrhagic complications are detected on a clinical MRI, there is uncertainty as to how the hemorrhage would have appeared on a CT scan. Conversely, MRI may aid in distinguishing angiographic contrast “staining” from blood in the subarachnoid space seen on CT. We sought to compare MRI‐ and CT‐based classification of HT and ICH in patients post‐EVT applying the HBC criteria to determine concordance across modalities and assess interrater agreement of both imaging modalities.

## Methods

The data that support the findings of this study are available from Lawrence L. Latour (latourl@ninds.nih.gov) upon reasonable request. The study was conducted as a retrospective analysis of de‐identified data established with the approval from the National Institutes of Health Office of Human Subjects Research (NIH OHSR Exempt #13285); determination of Not Human Subjects Research is based on the interpretation of 45 CFR 46 under “Research Involving Coded Private Information or Biological Specimens.”

### Patient Selection

Data from consecutive patients presenting to MedStar Washington Hospital Center (Washington, DC) and Suburban Hospital (Bethesda, MD) from April 1, 2018, to August 31, 2023, diagnosed with acute ischemic stroke and treated with EVT were selected for inclusion in this retrospective study, HECATE: Hemorrhage on CT versus MRI After Thrombectomy. The intention was to identify cases with both MRI and CT obtained close in time to permit comparison of findings. Data from patients were included if their post‐EVT imaging fulfilled the following criteria: (1) had MRI and CT ≤12 hours of each other OR (2) had CT bracketed by MRI pre‐ and post‐CT (ie, MRI‐CT‐MRI) all within 7 days post‐EVT. When MRIs were bracketed with multiple CTs in between, the latest CT and MRI scans were chosen. For cases with bracketed MRIs, the later MRI was used for the consensus read. If the HT grades differed between the bracketed MRIs, these cases were excluded from the intermodality analysis; however, both bracketed MRI reads were still used for the interrater agreement statistics. Patients with no CT and/or MRI post‐EVT were excluded. Of note, at our MRI‐based centers, EVT‐treated patients are primarily imaged with MRI post‐EVT unless there is a contraindication to MRI. Per our clinical management pathway, patients are routinely scanned with MRI early (2–6 hours) post‐EVT and again at 24 hours post‐EVT. Further, some patients are enrolled in an observational study that includes post‐EVT MRI scans at defined timepoints, including 5±2 days. Patients who receive a conventional noncontrast CT post‐EVT typically have that performed out of a concern for clinical deterioration due to hemorrhage or cerebral edema. Patients may also have a CT done to assess stability of known hemorrhage and/or safety to initiate anticoagulation. Specific clinical indications for any of the imaging obtained post‐EVT for individual patients was not collected.

### Imaging Protocol

CT images were performed for clinical purposes on multiple systems (Siemens and GE). Typical parameters included ∼32 to 36–5 mm thick axial‐oblique slices collected at 100–120 kVP and a 250 mm reconstruction diameter. MRI was performed for clinical and/or research purposes on Siemens 3T or Phillips 3T using a standardized clinical imaging protocol that included T2^*^ gradient recalled echo (T2^*^GRE) and fluid attenuated inversion recovery (FLAIR). All images were acquired in the axial oblique anterior commissure ‐ posterior commissure aligned plane, 220 mm field of view, 40–3.5 mm thick for T2^*^GRE and FLAIR contiguous interleaved slices. Relevant parameters are as follows: T2^*^GRE 320×240 matrix, 0.69 mm resolution, FA = 30 deg, 700–800/12 ms repetition time/echo time; and for the FLAIR 256/192 matrix, 0.86 mm resolution, FA = 150 deg, ∼6000/124/2020 ms repetition time/echo time/inversion time.

### Qualitative Image Analysis

Two experienced raters (A.W.H. and L.L.L.) blinded to patient identifiers, clinical information, and chronological order of the CT and MRI scans independently rated both HT and ICH subtype classification by evaluating T2^*^GRE MRI in combination with the diffusion‐weighted imaging and, separately, noncontrast CT with the HBC criteria.^4^ The interrater agreement statistics were based on these 2 experienced raters. Briefly, the HBC criteria (with ECASS definitions also provided in parentheses for reference) are provided in Table 1. Of note, the HBC HT grades of 1a, 1b, 1c, and 2 are mutually exclusive requiring the raters to choose only 1. However, the HBC grade 3 ICH subtypes are independent categories and were evaluated in addition to the HBC 1 and 2 HT grades, for example, 1 or more HBC 3 ICH subtypes could be selected in the presence or absence of an HBC 1 or 2 HT grade. The raters were provided the HBC criteria and were able to reference the published examples.^4^ HT class and any ICH subtypes identified were captured, for example, for a patient with HT class 1b and ICH subtype 3c (SAH), both were documented and included in the analysis. We did not apply the HBC criteria as an ordinal scale from HBC 2 to 3, that is, presence of any of the HBC 3 classes, for example, 3c (SAH), were not considered “worse” grades than HBC 2 (parenchymal hematoma grade 2 [PH‐2]). The raters performed the CT readings ∼2 weeks before the MRI readings to minimize recall bias. For discordant cases between the 2 raters, consensus was reached by a panel of readers (A.W.H., L.L.L., M.L., and S.S.) separately for both HT class and ICH subtypes. The consensus reads were used for intermodality analyses.

**Table 1:** Heidelberg Bleeding Classification Definitions

HBC class (ECASS definition)	Definition
0	No imaging evidence of hemorrhage
1a (HI‐1)	Hemorrhagic transformation of infarcted tissue limited to petechial hemorrhage
1b (HI‐2)	Hemorrhagic transformation of infarcted tissue involving area(s) of confluent petechiae
1c (PH‐1)	Parenchymal hematoma occupying ≤30% of the infarcted tissue without substantive mass effect
2 (PH‐2)	Parenchymal hematoma occupying >30% of the infarcted tissue with substantive mass effect

ICH subtype	Definition
3a (IPH)	Remote intraparenchymal hemorrhage
3b (IVH)	Intraventricular hemorrhage
3c (SAH)	Subarachnoid hemorrhage
3d (SDH)	Subdural hemorrhage

ECASS indicates European Cooperative Acute Stroke Study; HI‐1, hemorrhagic infarction grade 1; HI‐2, hemorrhagic infarction grade 2; ICH, intracranial hemorrhage; IPH, intraparenchymal hemorrhage; IVH, intraventricular hemorrhage; PH‐1, parenchymal hematoma grade 1; PH‐2, parenchymal hematoma grade 2; SAH, subarachnoid hemorrhage; and SDH, subdural hemorrhage.

### Clinical Outcomes

The rate of symptomatic intracranial hemorrhage (sICH) was reported using the SITS‐MOST criteria, a worsening at 24‐hour National Institutes of Health Stroke Scale score by ≥ 4 and HT grade 2 (PH‐2). The rate of asymptomatic ICH was also reported based on the presence of any HT and/or any ICH subtype that did not meet the sICH criteria. Additional clinical outcomes reported were 24‐hour National Institutes of Health Stroke Scale, discharge to home, modified Rankin scale score at 30–90 days (90 days was used unless only 30 days was available), and favorable outcome defined as modified Rankin scale score 0–2 at 30–90 days.

### Statistical Analysis

Study patient characteristics including demographic, clinical, acute intervention, and outcome data were summarized. Variables were reported as median (interquartile range 25–75) or total number (percentage, including 95% CI) as appropriate. The concordance of the HBC classifications including presence of HT, HT class, and ICH subtypes were compared between CT and MRI based on the consensus reads. The frequencies of classifications for CT, MRI, and both imaging modalities were reported. Chi‐square test comparing frequencies was used with significant differences based on *P*<0.05. Interrater agreement statistics were calculated using 2 independent reads performed by raters (A.W.H. and L.L.L.), both with extensive experience in reading CT and MRI in the context of acute intervention.^6^ Interrater agreement for each HBC classification including presence of HT, HT class, and ICH subtypes was assessed using unweighted Cohen's kappa (ĸ) including 95% CI. Cohen's ĸ value ranges of 0.01–0.20, 0.21–0.40, 0.41–0.60, 0.61–0.80, and 0.81–0.99 indicated poor, fair, moderate, substantial, and excellent agreement. IBM SPSS Statistics v19.0 was used for all statistical analyses performed.

## Results

### Patient Selection

Of the 326 patients treated with EVT during the study period, 87 patients (27%) met the study criteria. Patients were excluded due to following reasons: 114 had CT and MRI at follow‐up but were acquired >12 hours from each other, 105 had only CT or MRI at follow‐up, 7 had insufficient follow‐up for evaluation due to image quality, and 13 were excluded from the intermodal analysis due to differing HT grades for the bracketed MRI scans.

The 87 study patients had a median age of 68 years [60–74], 54% (n = 47) female, admission National Institutes of Health Stroke Scale score of 18 [13–23], preadmission modified Rankin scale score 0 [0‐0], onset time (calculated as last seen normal to triage) of 155 minutes [72–382], and 47% (n = 41) were treated with intravenous or intraarterial thrombolysis (Table 2). The EVT procedural details are listed in Table 2; of note, successful recanalization (modified Thrombolysis in Cerebral Infarction 2b/3) was achieved in 93% (77/83) of patients with a median onset to recanalization time of 297 minutes [217–552]. The rate of sICH was 4.6% (4/87, 95% CI [1.3–11.4%]; *P* = 1.0) across both imaging modalities (2/4 cases were concordant between CT and MRI and 1 additional case for each CT and MRI also met the sICH criteria). The rate of asymptomatic ICH across both imaging modalities was 40% (35/87, 95% CI [30%–51%]). The rates of asymptomatic ICH based on CT and MRI were 44% (38/87, 95% CI [33%–55%]) and 52% (45/87, 95% CI [41%–63%]); *P* = 0.29, respectively. Additional clinical outcomes included median 24‐hour National Institutes of Health Stroke Scale score of 17 [11–25], 15% (n = 13) of patients were discharged to home, median latest available (30–90 days) modified Rankin scale score of 4,^2^, ^3^, ^4^, ^5^, ^6^ and 28% (21/76) achieved favorable clinical outcome (Table 2).

**Table 2:** Patient Characteristics of Study Group

Variable	All patients (n=87)	
**Baseline characteristics**		
Age, y	68 [60–74]	
Female sex	47 (54%)	
Onset (last seen normal to triage time, min)	155 [72–382]	
IV/IA thrombolysis	41 (47%)	
Anterior circulation	79 (91%)	
Target occlusion site	eICA	2 (2%)
	iICA	20 (23%)
	M1	38 (44%)
	M2	18 (21%)
	M3	1 (1%)
	Basilar	8 (9%)
Preadmission mRS score	0 [0–0]	
Admission NIHSS score	18 [13–23]	
**EVT procedural details**		
Onset to recanalization (min)	297 [217–552]	
General anesthesia used in EVT	83 (95%)	
Multiple passes^*^	48/80 (60%)	
mTICI^*^	0	3/83 (4%)
	1	1/83 (1%)
	2a	2/83 (2%)
	2b	44/83 (53%)
	3	33/83 (40%)
Successful recanalization^*^	77/83 (93%)	
**Clinical outcomes**		
Symptomatic ICH	4 (4.6%)	
Asymptomatic ICH	35 (40%)	
24 h NIHSS	17 [11–25]	
Discharge to home	13 (15%)	
mRS at 30 or 90 d^†^	4 [2–6]	
Favorable outcome (mRS 0–2)^‡^	21/76 (28%)	

EVT indicates endovascular therapy; IA, intraarterial; ICH, intracranial hemorrhage; IV, intravenous; mRS, modified Rankin scale; mTICI, modified Thrombolysis in Cerebral Infarction; and NIHSS, National Institutes of Health Stroke Scale.

Denominator for the percentage calculations is 87 unless otherwise indicated.

* Endovascular device was not deployed in 4 patients; number of passes data were not available in 3 additional patients with device deployed.

^†^ mRS score was used from 90 days if available otherwise mRS score at 30 days was used.

^‡^ mRS score was not available in 11 patients at either 30 or 90 days to determine outcome.

### Imaging Scans

For the intermodality analysis, 25 patients had CT with bracketed MRIs and another 21 had CT done before a nonbracketed MRI, resulting in 46 total of 87 (53%) with CT done before MRI. In these 46 patients, there was a median time of 10 hours [6–24] in between scans. There were 41 of 87 (47%) of patients with nonbracketed MRI done before CT, median time was 7 hours^4^, ^5^, ^6^, ^7^, ^8^, ^9^ in between scans.

### CT and MRI Concordance

**Figure 1:**
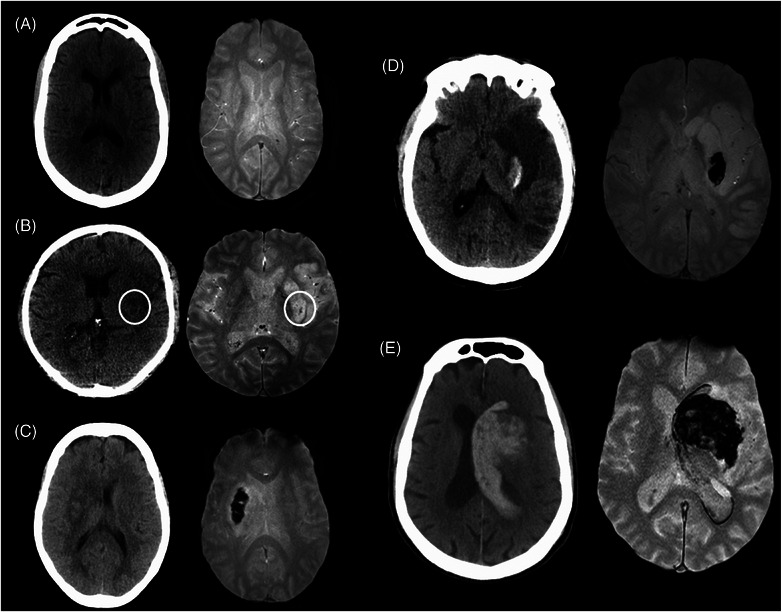
**Examples of concordant Heidelberg Bleeding Classification of hemorrhagic transformation between CT and MRI**. **A**, HBC 0: 63‐year‐old female, admission NIHSS score 7, basilar artery (mTICI 2b), CT 7 hours before MRI. **B**, HBC 1a: 74‐year‐old male, admission NIHSS score 20, intravenous alteplase, M1 MCA (mTICI 3), CT 2 hours before MRI. **C**, HBC 1b: 61‐year‐old female, admission NIHSS score 15, M1 MCA (mTICI 2b), CT 1 hour after MRI. **D**, HBC 1c: 73‐year‐old male, admission NIHSS score 23, iICA (mTICI 3), CT 7 hours after MRI. **E**, HBC 2: 74‐year‐old male, admission NIHSS score 28, iICA (mTICI 2b), CT 6 hours before MRI. CT indicates computed tomography; HBC, Heidelberg Bleeding Classification; iICA, intracranial internal carotid artery; MCA, middle cerebral artery; MRI, magnetic resonance imaging; mTICI, modified Thrombolysis in Cerebral Infarction; and NIHSS, National Institutes of Health Stroke Scale.

We found a 68% (59/87, 95% CI [57%–77%]) agreement overall between CT and MRI for hemorrhage classification post‐EVT. The presence of hemorrhage was detected on at least 1 modality in 60% (52/87 95% CI [49%–70%]) of patients, with details of the different numbers and rates seen on CT and/or MRI provided in Table 3. Any hemorrhage was detected in 54% (47/87 95% CI [43%–65%]) of patients on MRI versus 46% (40/87 95% CI [35%–57%]) on CT (*P* = 0.29). Based on the HBC HT classes, 1a–2, there was intermodality agreement for 42% (20/48 95% CI [28%–57%]) of patients (Table 3). Figure 1 illustrates patients with concordant HBC HT classes on both CT and MRI. Of note, difference in HT severity was balanced between imaging modalities for the cohort of nonbracketed MRI done before CT, with 17% (7 of 41) of patients with worse CT HT grade and the same number with worse MRI HT grade. Overall, in this cohort of 41 patients, the CT and MRI HT grades were concordant in 66% (27/41).

**Table 3:** Contingency Table Comparing CT Versus MRI Reads for HT Grades Based on Consensus Reads

Consensus reads % (n)	CT HT grade						
**MRI HT grade**		None	1a (HI‐1)	1b (HI‐2)	1c (PH‐1)	2 (PH‐2)	Total patients
	None	45% (39)^*^	0% (0)	1% (1)	0% (0)	0% (0)	46% (40)
	1a (HI‐1)	6% (5)	2% (2)	2% (2)	1% (1)	0% (0)	11% (10)
	1b (HI‐2)	8% (7)^†^	2% (2)	8% (7)	2% (2)	0% (0)	21% (18)
	1c (PH‐1)	0% (0)	0% (0)	5% (4)	3% (3)	2% (2)	10% (9)
	2 (PH‐2)	0% (0)	0% (0)	1% (1)	1% (1)	9% (8)	11% (10)
	Total patients	59% (51)	5% (4)	17% (15)	8% (7)	11% (10)	100% (87)

1a (HI‐1) indicates hemorrhagic infarction grade 1; 1b (HI‐2), hemorrhagic infarction grade 2; 1c (PH‐1), parenchymal hematoma grade 1; 2 (PH‐2), parenchymal hematoma grade 2; CT, computed tomography; HT, hemorrhagic transformation; ICH, intracranial hemorrhage; MRI, magnetic resonance imaging; and SAH, subarachnoid hemorrhage.

* There were 4 patients included in the total HT grade as none but who had an ICH subtype: 3 patients where SAH was read by consensus on CT and 1 where remote IPH only was read on CT.

^†^ There were 2 patients included with ICH subtype SAH on CT with 1b HT grade on MRI.

**Figure 2:**
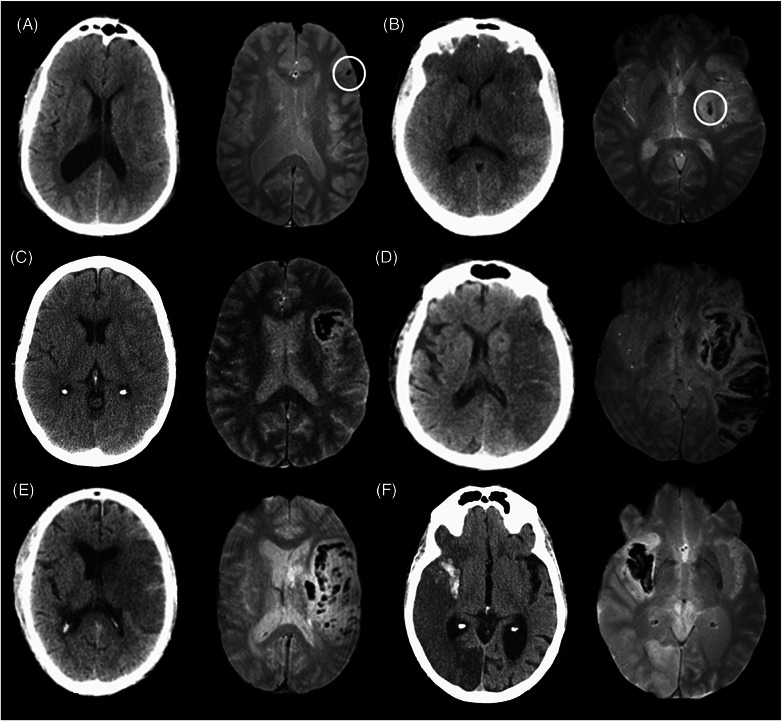
**Examples of discordant Heidelberg Bleeding Classification of hemorrhagic transformation and intracranial hemorrhage between CT and MRI**. **A**, HBC 0 on CT, 1a on MRI: 57‐year‐old male, admission NIHSS score 23, M2 MCA (mTICI 3), CT 10 hours before MRI. **B**, HBC 0 on CT, 1a on MRI: 36‐year‐old female, admissionNIHSS score 28, IV alteplase, M2 MCA (mTICI 2a), CT 9 hours after MRI. **C**, HBC 0 on CT, 1b on MRI: 69‐year‐old female, admission NIHSS score 17, IV alteplase, M2 MCA (mTICI 2b), CT <1 hour before MRI. **D**, HBC 1a on CT, 1b on MRI: 75‐year‐old male, admission NIHSS score 26, IV alteplase, M1 MCA (mTICI 3), CT 10 hours before MRI. **E**, HBC 3c (SAH) on CT, 1b on MRI: 61‐year‐old male, admission NIHSS score 17, eICA (mTICI 2b), CT 8 hours before MRI. **F**, HBC 1c on CT, 1c and 3C (SAH) on MRI: 62‐year‐old male, admission NIHSS score 25, M1 MCA (mTICI 2b), CT 1 hour after MRI. CT indicates computed tomography; eICA, extracranial internal carotid artery; HBC, Heidelberg Bleeding Classification; IV, intravenous; MCA, middle cerebral artery; MRI, magnetic resonance imaging; mTICI, modified Thrombolysis in Cerebral Infarction; NIHSS, National Institutes of Health Stroke Scale; and SAH, subarachnoid hemorrhage.

Discordance between modalities occurred primarily in the identification of scattered small petechial (Figure 2A, B) and confluent petechial (Figure 2C–E) hemorrhage. The amount and confluence of petechial hemorrhage were more pronounced on MRI compared with CT as demonstrated in Figure 2. T2^*^GRE MRI tended to have increased sensitivity to scattered petechial hemorrhage (HBC 1a) as compared with CT with 17% (2/12) intermodal agreement (Table 3). The interrater agreement (Table 5) of HBC class 2 (ie, PH‐2) was substantial for MRI (ĸ = 0.781) and excellent in CT (ĸ = 0.951), with 67% (8/12) intermodal agreement (*P* = 0.004) (Table 3).

There was some intermodality discordance on the presence of ICH subtypes, with details of the different numbers and rates on CT and/or MRI provided in Table 4. SAH was detected in 24% (21/87, 95% CI [16%–35%]) of patients on CT and/or MRI with 29% (6/21) intermodal agreement (Table 4). The distinction of HBC class 1b and/or SAH including involvement of/around the sylvian fissure was particularly challenging (Table 3, Figure 2E, F). There were no detected cases of subdural hemorrhage HBC Class 3d (SDH) on either modality.

**Table 4:** Contingency Table Comparing CT Versus MRI Reads for ICH Subtypes Based on Consensus Read

Consensus read^*^ (%) (n)	CT ICH subtype			
**MRI ICH subtype**		None	3a (Remote IPH)	Total patients
	None	98% (85)	1% (1)	99% (86)
	3a (Remote IPH)	1% (1)	0% (0)	1% (1)
	Total patients	99% (86)	1% (1)	100% (87)
			
		None	3b (IVH)	Total patients
	None	90% (78)	2% (2)	92% (80)
	3b (IVH)	2% (2)	6% (5)	8% (7)
	Total patients	92% (80)	8% (7)	100% (87)
			
		None	3c (SAH)	Total patients
	None	76% (66)	10% (9)	86% (75)
	3c (SAH)	7% (6)	7% (6)	14% (12)
	Total patients	83% (72)	17% (15)	100% (87)

3a (remote IPH) indicates remote intraparenchymal hemorrhage; 3b (IVH), intraventricular hemorrhage; 3c (SAH), subarachnoid hemorrhage; 3d (SDH), subdural hemorrhage; CT, computed tomography; ICH, intracranial hemorrhage; IPH, intraparenchymal hemorrhage; IVH, intraventricular hemorrhage; MRI, magnetic resonance imaging; SAH, subarachnoid hemorrhage; and SDH, subdural hemorrhage.

* There were no reported cases of 3d (SDH) based on consensus read.

No hemorrhage was detected in 52% (45/87) of patients on CT and 44% (38/87) on MRI (*P* = 0.29). On CT, 24% (21/87), and on MRI, 36% (31/87), of patients had HT grade 1a, 1b, or 2 without any ICH subtype present (*P* = 0.09). There were 17% (15/87) of patients on CT and 18% (16/87) on MRI that had HT grade 1a, 1b, or 2, in addition to one or more ICH subtypes (*P* = 0.86). Only 7% (6/87) and 2% (2/87) of patients on CT and MRI, respectively, had an ICH subtype with HT grade of none (*P* = 0.11). Almost all of these cases, 88% (7/8) had subtype 3c (SAH) only, with the exception of 1 remote intraparenchymal hemorrhage.

### Interrater Agreement

Quantitative results demonstrated that MRI had the best interrater agreement for the classification of HBC 0 (no hemorrhage) with excellent concordance (ĸ = 0.882, 95% CI [0.843–0.916]) compared with substantial agreement for CT (ĸ = 0.683, 95% CI [0.564–0.775]) (*P* = 0.005) (Table 5). HBC 1a had poor agreement on CT (ĸ = 0.113, 95% CI [−0.078 to 0.306]) although moderate on MRI (ĸ = 0.442, CI95% [0.329–0.589]) (*P* = 0.20). HBC 1c was challenging in part due to difficulties in determining the extent of hemorrhage within the infarcted tissue on diffusion‐weighted imaging compared with CT (Figure 2). Discordance was also introduced in assessing mass effect when the infarcted tissue included the basal ganglia (Figure 2E). In contrast, the interrater agreement of HBC class 2 (PH‐2) was substantial for MRI (ĸ = 0.781, CI95% [0.709–0.840]) and excellent in CT (ĸ = 0.951, 95% CI [0.929–0.967]) (*P* = 0.004) (Table 5, Figure 1E).

**Table 5:** Interrater Agreement for CT and MRI Across Presence of Hemorrhage, HT Grade, and ICH Subtypes

Interrater agreement for MRI and CT								
	None	1a	1b	1c	2	3a	3b	3c
**MRI**								
Unweighted Cohen's ĸ	0.882	0.442	0.55	0.425	0.781	0.486	0.638	0.645
95% CI	[0.843–0.916]	[0.329–0.589]	[0.425–0.657]	[0.283–0.554]	[0.709–0.840]	[0.351–0.605]	[0.537–0.732]	[0.544–0.737]
**CT**								
Unweighted Cohen's ĸ	0.683	0.113	0.51	0.73	0.951	0.492	0.808	0.641
95% CI	[0.564–0.775]	[−0.078–0.306]	[0.367–0.652]	[0.634–0.815]	[0.929–0.967]	[0.335–0.631]	[0.734–0.870]	[0.531–0.756]

1a indicates hemorrhagic infarction grade 1; 1b, hemorrhagic infarction grade 2; 1c, parenchymal hematoma grade 1, 2, parenchymal hematoma grade 2; 3a (remote IPH), remote intraparenchymal hemorrhage; 3b (IVH), intraventricular hemorrhage; 3c (SAH), subarachnoid hemorrhage; 3d (SDH), subdural hemorrhage; CT, computed tomography;HT, hemorrhagic transformation; ICH, intracranial hemorrhage; IPH, intraparenchymal hemorrhage; IVH, intraventricular hemorrhage; MRI, magnetic resonance imaging; SAH, subarachnoid hemorrhage; and SDH, subdural hemorrhage.

## Discussion

We found a 68% (59/87, 95% CI [57%–77%]) agreement overall between CT and MRI for classification of hemorrhage after EVT. Where there was discordance, MRI (T2^*^GRE) tended to have an increased sensitivity to petechial hemorrhage as compared with CT, overall consistent with prior studies by Arnould et al and Renou et al.^7^, ^8^ Though at a relatively low frequency in our EVT‐treated patients, our finding of hemorrhage classification crossing from petechial HT on CT to parenchymal hematoma on MRI occurred at a higher frequency than reported in prior studies of intravenous tissue plasminogen activator‐treated patients; in Renou et al, no discordant HT classification crossed these categories.^7^, ^8^ Reassuringly for its clinical relevance, there was still excellent agreement for HBC class 2 (ie, PH‐2) between MRI and CT. To our knowledge, this is the first MRI versus CT comparison of post‐EVT hemorrhage classification applying the HBC, including evaluation of the HBC class 3 ICH subtypes (remote intraparenchymal hematoma, intraventricular hemorrhage, SAH, subdural hematoma). For these HBC class 3 ICH categories, T2^*^GRE and CT were overall comparable in the identification of remote intraparenchymal hematoma and intraventricular hemorrhage. For class 3c (SAH), there was discordance for ∼20% of cases in both directions for CT and MRI detecting “SAH.” For imaging identification of SAH, there is the known complicating factor of iodinated contrast extravasation mimicking SAH.^9^ We found this to be an issue in our post‐EVT cohort with 9 (10%) patients identified as having SAH on CT without any hemorrhage on T2^*^GRE MRI. FLAIR was not used for study image interpretation. On subsequent review, the majority of these 9 cases were positive for SAH on FLAIR, but the interpretation of SAH on postgadolinium FLAIR is complicated by the presence of hyperintense acute reperfusion marker. Anecdotal comments from the blinded raters noted the difficulty in discerning iodinated contrast versus SAH while performing their image interpretations of CT (Figure 2). We did not perform Hounsfield unit analysis on the post‐EVT CT scans to distinguish contrast staining versus SAH.^10^

We found variability in interrater agreement across categories for identifying HT or ICH post‐EVT on T2^*^GRE MRI and CT. Notable findings included a higher inter‐rater agreement with MRI than CT for scattered petechial HT, whereas it was higher with CT than MRI for classification of parenchymal hematoma. Anecdotal comments from the blinded raters noted difficulty in applying the current definitions of the HI and PH classifications and coming to agreement, specifically those that do not fit exactly into HBC class 1b, 1c, or 2, for example, a basal ganglia hemorrhage that appears borderline on MRI for confluent petechiae versus hematoma and that occupied most of the infarcted tissue; or a hemorrhage that occupies <30% of the infarct but seems to have associated edema with substantive mass effect (Figure 2). These qualitative observations are consistent with those noted by Renou et al and the meta‐analysis performed by Guenego et al when reporting on the application of the ECASS classification criteria.^8^, ^11^ The size of the overall infarct is more clearly visualized on MRI than on CT, so the proportion of the infarct that is occupied by hematoma is more clearly delineated on MRI.^12^, ^13^ Surrounding edema is also more clearly seen on MRI than CT, but interpretation is required as to whether the edema is due to the hematoma or to the infarct itself.

Our study has several limitations. First, this study cohort is biased toward patients with more severe neurologic deficit, as reflected in only approximately one quarter having a favorable outcome at 30–90 days. (In comparison, 37% of the 326 total patients treated with EVT during this study period had a favorable outcome at 30–90 days.) Brain imaging, particularly CT, was driven by clinical indications and not done systematically and concurrent with MRI. Also, study patients with the CT bracketed by MRI had a longer duration between modalities than those included with CT‐MRI paired within 12 hours. To minimize any potential evolution of hemorrhage between scans for those included with CT bracketed by MRI, only those with consistent MRI HBC ratings were included in the intermodal analysis. Second, we identified small numbers of patients with each of the individual HBC class 3 subtypes of ICH, with no patients identified as having subdural hemorrhage by consensus read, which limits the intermodality and interrater comparisons. Still, our findings with regard to SAH detection and SAH mimic are noteworthy and warrant further evaluation in larger data sets. Third, for the purposes of this systematic, blinded image interpretation, we used only the diffusion‐weighted imaging and T2^*^GRE for the MRI sequences. In contrast, for both clinical trials and routine clinical applications, all available MRI sequences including FLAIR would be evaluated, which we expect would further increase the sensitivity and specificity of SAH identification by MRI post‐EVT, particularly if gadolinium has not been administered for a prior MRI for the index event. We also did not use SWI, which is used at some centers rather than GRE. SWI is more sensitive to blood products than GRE^14^ and may result in further intermodality and interrater differences in hemorrhage ratings. Finally, our centers did not use dual energy CT during this study period, a technique increasingly utilized at centers to distinguish iodinated contrast from SAH in patients post‐EVT.^15^, ^16^ We would expect an increased comparability, and accuracy, in the HBC class 3c (SAH) category for MRI and CT if dual energy CT were used as the comparator.

As we described, we did encounter difficulties in categorizing post‐EVT hemorrhage, either on CT or on MRI, even using the most recently developed HBC criteria, that may have implications in safety outcome and/or clinical management, particularly if one has minimal petechial HT versus confluent petechial HT or parenchymal hematoma. In hemorrhage following intravenous thrombolytics, only parenchymal hematoma has been associated with neurologic worsening.^8^, ^17^, ^18^ Similarly, Honig et al found that post‐EVT, PH‐2 on 24‐hour CT was associated with unfavorable clinical outcome and was the only type of hemorrhage, applying ECASS‐3 criteria, that led to symptomatic ICH; they found no association between petechial HT as a group (hemorrhagic infarction grade 1 [HI‐1] and grade 2 [HI‐2] combined) and outcome.^19^ Of note, ECASS‐3 criteria for sICH includes that the hemorrhage “was identified as the predominant cause of the neurologic deterioration.”^20^ However, in this study, we have observed that in many cases with HI‐2 (HBC 1b), there is extensive HT involvement of larger infarcts that can be seen post‐EVT as illustrated in Figure 2D–F. It would be difficult to distinguish whether it is the hemorrhage or the size of the infarct or edema associated with both the hemorrhage and the size that is accounting for neurologic outcome, and it is likely contributions from all of these factors. Prior studies may have categorized these patients as “asymptomatic ICH.” Further study is warranted in a larger patient sample to better understand the potential impact of extensive confluent petechial HT on clinical outcome in patients post‐EVT. There can also be difficulty in analyzing hemorrhage rates given the complexity of the hemorrhagic complications that can occur post‐EVT, for example, presence of HT plus intraventricular hemorrhage plus SAH. For this reason, we did not apply the HBC criteria as an ordinal scale and limit each scan to a single grade assuming any of the HBC 3 classes as the highest grade to assign. For example, the presence of any SAH (HBC 3c) is not necessarily a “worse” grade than a space‐occupying parenchymal hematoma (HBC 2).

In conclusion, our findings suggest that for patients post‐EVT, either imaging modality is reasonable to evaluate hemorrhage in clinical or research applications provided reliable detection of scattered small petechiae (HBC class 1a) is not needed. Also, if CT is to be used in this setting, dual energy CT would be preferred over standard noncontrast CT to distinguish SAH more reliably from concomitant iodinated contrast that may persist. The challenges we experienced in systematically applying the HBC to post‐EVT hemorrhage suggest a need to further revise/develop a hemorrhage classification system using an ordinal scale for EVT‐treated patients. Our findings could serve as a basis for a new scoring system that takes into consideration the differences between hemorrhage rating on MRI and CT demonstrated in this study. This image data set could also be used as a future training tool for both clinical and research applications.^21^ Further work is also needed to understand the implications of the different types of hemorrhage post‐EVT on clinical outcomes.

## Sources of Funding

Financial support for this work was provided by the Intramural Research Program of the National Institute of Neurological Disorders and Stroke, National Institutes of Health.

## Disclosures

None of the authors have anything to disclose.
